# Roles of Nuclear Receptors in Vascular Calcification

**DOI:** 10.3390/ijms22126491

**Published:** 2021-06-17

**Authors:** Giulia Chinetti, Jaap G. Neels

**Affiliations:** 1 Université Côte d’Azur, CHU, INSERM, C3M, 06204 Nice, France; Giulia.CHINETTI@univ-cotedazur.fr; 2 Université Côte d’Azur, INSERM, C3M, 06204 Nice, France

**Keywords:** transcription factors, gene regulation, mineralization, calcium, extracellular matrix, arterial wall, vascular cells

## Abstract

Vascular calcification is defined as an inappropriate accumulation of calcium depots occurring in soft tissues, including the vascular wall. Growing evidence suggests that vascular calcification is an actively regulated process, sharing similar mechanisms with bone formation, implicating both inhibitory and inducible factors, mediated by osteoclast-like and osteoblast-like cells, respectively. This process, which occurs in nearly all the arterial beds and in both the medial and intimal layers, mainly involves vascular smooth muscle cells. In the vascular wall, calcification can have different clinical consequences, depending on the pattern, localization and nature of calcium deposition. Nuclear receptors are transcription factors widely expressed, activated by specific ligands that control the expression of target genes involved in a multitude of pathophysiological processes, including metabolism, cancer, inflammation and cell differentiation. Some of them act as drug targets. In this review we describe and discuss the role of different nuclear receptors in the control of vascular calcification.

## 1. Introduction

### 1.1. Nuclear Receptors

**Figure 1:**
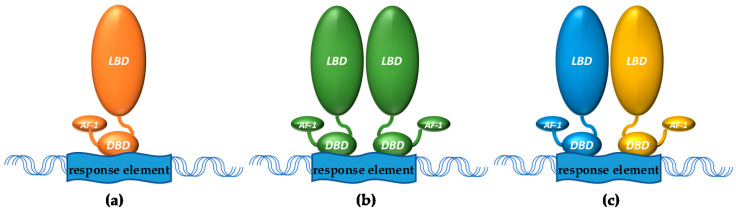
NRs can bind as (**a**) monomers (e.g., certain orphan receptors), (**b**) homodimers (e.g., steroid hormone receptors), or (**c**) heterodimers (often with RXR) to DNA response elements. LBD = ligand binding domain, DBD = DNA binding domain, AF-1 = activation function 1.

There are seven different subfamilies (NRo to NR6) of nuclear receptors (NRs) that together form a large superfamily (48 members in humans) sharing similar structures: An activation function (AF)-1 region at the N-terminus, followed by a DNA-binding domain recognizing specific response elements that is connected through a hinge region to a C-terminal AF-2 ligand binding domain [1]. NRs can repress or activate gene transcription as monomers, homodimers, or heterodimers (Figure 1, the latter often with retinoid X receptors (RXRs)) as part of a larger complex. In absence of ligand, these transcriptional complexes often contain co-repressor proteins and repress gene expression, and upon ligand binding, co-activator proteins can replace these co-repressors through a ligand-induced conformational change to form a co-activator complex that initiates gene transcription. Many NRs also exhibit non-genomic activities, for example modulation of signaling pathways, through which they can indirectly affect the activity of other transcription factors. Similarly, by binding directly to them, NRs can trans-suppress the activity of other transcription factors. Their expression can be influenced by specific miRNAs [2,3] as well as by inflammatory stimuli [4,5]. Moreover, transcriptional activity and/or stability of NRs can be modified by post-translational modifications, including phosphorylation, acetylation, methylation and SUMOylation [6,7]. This narrative review focuses on the role of different NRs, for which adequate data are available, in vascular calcification.

### 1.2. Vascular Calcification

#### 1.2.1. Generalities

**Figure 2:**
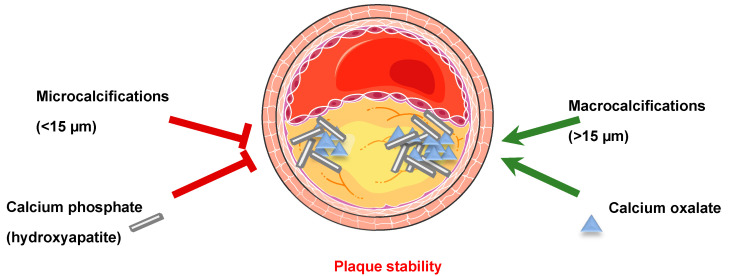
Vascular calcification affects plaque stability. Microcalcifications (0.5–15 µm) and spotty calcifications are positively associated with plaque rupture. Plaques with macrocalcifications (>15 µm) are more often asymptomatic, suggesting that large calcifications are beneficial for plaque stability. The nature of calcium minerals composing calcifications also plays a role in plaque stability. Calcifications composed of hydroxyapatite (calcium phosphate) crystals are associated with plaque instability, while calcium oxalate containing calcifications, present in approximately 30% of the human atherosclerotic plaques, are mainly associated with plaque stability.

Vascular calcification (VC) is defined as an inappropriate deposition of calcium minerals occurring in nearly all arterial beds, in both the intima and in the media. Medial calcification, often observed in diabetic or chronic kidney disease (CKD) patients [8], is characterized by diffuse mineral deposition throughout the vasculature that can occur completely independently of atherosclerosis or in parallel, and it is commonly observed in femoral, tibial, and uterine arteries. Among non-dialyzed CKD patients, more than 50% have coronary artery calcification, whereas 70–90% of prevalent dialysis patients have significant coronary artery calcification. Histological studies comparing deceased dialyzed patients to non-CKD patients that died from a coronary event showed that dialyzed patients had more calcified atherosclerotic plaques, but not more plaques [9]. Intimal calcification is associated with inflammation and the development of atherosclerotic occlusive lesions, while adjacent regions of the vessel wall may remain remarkably normal. This intimal calcification is an indicator of an advanced stage of atherosclerosis and is observed in the aorta, coronary arteries, and carotids [10,11]. VC reduces aortic and arterial elasticity, impairs cardiovascular hemodynamics [12], thus contributing to hypertension, aortic stenosis and cardiac hypertrophy [13,14,15]. However, atherosclerotic plaque stability depends on the differential amounts, sizes, shapes and positions of calcification. Indeed, microcalcifications (0.5–15 µm) and spotty calcifications represent an early stage of VC and are positively associated with plaque rupture [16]. The local stress produced by microcalcifications in the fibroatheroma cap depends on the material tissue properties, their spacing and their alignment relative to the tensile axis of the cap [17]. The continuous coalescence of microcalcifications transforms them into large calcifications of distinct geometries. Since patients presenting atherosclerotic plaque macrocalcifications (>15 µm) are more often asymptomatic, the hypothesis that large calcifications are beneficial for plaque stability is widely accepted [18,19,20] (Figure 2). Indeed, the presence of macrocalcifications in human carotid lesions correlated with a transcriptional profile characteristic of stable plaques, characterized by an altered smooth muscle cells (SMC) phenotype and extracellular matrix (ECM) composition and repressed inflammation [21].

Moreover, the nature of calcium minerals composing calcifications also plays a role in plaque stability [22,23]. Indeed, a positive association is observed between calcifications composed by hydroxyapatite crystals (HA, calcium phosphate) and plaque instability, while calcium oxalate containing calcifications, present in approximately 30% of human atherosclerotic plaques, are mainly associated with plaque stability [23] (Figure 2).

Interestingly, although at first look atherosclerosis and osteoporosis appear as two independent situations, several epidemiological studies reveal an unquestionable link between these two pathologies, suggesting the involvement of similar pathophysiological mechanisms [24]. Indeed, clinical studies demonstrated that patients with cardiovascular diseases (CVD) present lower bone mineral density and faster bone loss [25]. Particularly, VC appears to be a central event linking CVD and bone loss. Indeed, VC is associated with a higher risk of vertebral fractures in menopausal females [26]. Moreover, osteoporosis relates to increased CVD such as atherosclerosis and myocardial infarction [24]. This association cannot only be explained by age or common risk factors, such as smoking, alcohol, diabetes, physical activity and menopause, but probably implies common metabolic pathways and cell-mediated mechanisms [27], among which is inflammation [25].

Passive calcification can be induced by hyperphosphatemia and/or hypercalcemia and initiates mineralization by depositing calcium and phosphate ions on the organic ECM. On the other hand, active calcification is a dynamic cellular-dependent process sharing many features with bone formation.

#### 1.2.2. Bone Mineralization

Bone is a specialized connective tissue consisting of cells and mineralized extracellular matrix. Organic and inorganic components of the matrix are mainly formed by collagen I fibers and spindle- or plate-shaped crystals of HA, respectively. Bone contains osteoblasts (OTBs) originating from local mesenchymal stem cells, responsible for the production of the calcified matrix [28] and expressing specific markers such as tissue-nonspecific alkaline phosphatase (TNAP/ALP), bone morphogenetic protein (BMP) and the Runx family of transcription factors (Runx-1 and Runx-2).

TNAP/ALP secreted by OTBs hydrolyzes both ATP and inorganic pyrophosphate (PPi), providing phosphate to promote mineralization [29]. Moreover, the mesenchymal origin of OTB has been challenged by the fact that expression of TNAP/ALP increases upon monocyte differentiation into macrophages [30]. Intriguingly, a population of calcifying cells of myeloid origin (MCC) has been described [31], belonging to the monocyte/macrophage lineage and expressing CD14 and CD68, as well as two OTB markers, osteocalcin and TNAP/ALP. MCC can be differentiated from peripheral blood mononuclear cells and promote atherosclerotic calcification in vivo in ApoE^−/−^ mice, by paracrine mechanisms [32]. In human, MCC are abundant in the neointima of calcified carotid atherosclerotic lesions from diabetic patients [31].

Moreover, OTBs are also necessary for osteoclast (OTC) differentiation from hematopoietic stem cells, by expressing M-CSF (macrophage colony-stimulating factor) and RANKL (receptor for activation of nuclear factor kappa-B (NF-κB) ligand), [33], a trans-membrane ligand binding to its receptor RANK, present on the membranes of monocytes driving OTC differentiation by increasing expression of tartrate-resistant acid phosphatase (TRAP) [34].

#### 1.2.3. Soft Tissue Calcification

Physiological mineralization occurs in hard tissues (bones), whereas pathological calcification occurs in soft tissues, following mechanisms similar to those observed in bone metabolism. In blood vessels, ectopic bone formation involves many different cell types, including vascular SMC (VSMC) and macrophages and key osteogenic signals regulating vascular calcium phosphate homeostasis [35]. Macrophage-derived cytokines (IL-1β, IL-6, IL-8, TNFα) induce osteogenic differentiation and mineralization of VSMC [36]. The production of 1,25(OH)_2_D_3_ (vitamin D_3_) by macrophages in response to microenvironment factors, such as oxidized LDL and IFNγ, promotes trans-differentiation of VSMC to OTBs by increasing expression and/or activity of TNAP/ALP [37,38]. Co-localization of the macrophage marker CD68 with carbonic anhydrase type II (CA2) has been observed in human atherosclerotic plaques [39] where TRAP-positive multinucleated giant OTC-like cells have also been found [40], thus providing evidence for the existence of OTC-like cells within vascular walls.

## 2. Role of Nuclear Receptors in Vascular Calcification

### 2.1. Vitamin D Receptor (VDR)

Vitamin D is synthesized by the skin upon exposure to sunlight or can be orally ingested through the diet [41]. In the skin, 7-dehydrocholesterol is transformed into cholecalciferol by ultraviolet B radiation. This cutaneously synthesized cholecalciferol and the ingested mix of ergocalciferol and cholecalciferol, bound to vitamin D binding protein (DBP), are transported to the liver where they are hydroxylated by 25-hydroxylases to 25-hydroxy vitamin D (25(OH)D), which is then further hydroxylated in the kidneys by 1-α hydroxylase to produce the physiologically active hormone 1,25-dihydroxyvitamin D (1,25(OH)_2_D) [41]. 25(OH)D and 1,25(OH)_2_D can be inactivated by 24-hydroxylase. Upon subsequent release in the circulation, 1,25(OH)_2_D can exerts its effects in different target tissues. These can be non-genomic and genomic effects, but in this review we focus on the genomic effects involving VDR, which upon binding of 1,25(OH)_2_D heterodimerizes with RXR. After translocating to the nucleus, this heterodimer induces transcription of target genes by binding to vitamin D response elements in their promoter regions. Over 11,000 putative VDR target genes have been identified that are known to control different mechanisms varying from cell adhesion and metabolism to tissue differentiation, development and angiogenesis [42].

For close to a century, excess vitamin D intake, resulting in supraphysiological levels of vitamin D, has been a known cause of VC and excess administration of vitamin D is widely used in experimental animal models for calcification of different organs including the vasculature [43]. However, insufficient or deficient vitamin D status have also been associated with VC. Low dietary vitamin D has been shown to lead to VC in mice, but the degree of VC was less than caused by excess vitamin D [44,45]. Together, these results suggest a U-shaped dualistic role of vitamin D action promoting VC in both low and high concentrations.

Several mouse studies have investigated the role of the VDR in VC with varying results. Schmidt et al. observed an increase in aortic calcification accompanied by an increase in osteogenic gene expression in aortic roots of VDR^−/−^ mice [46]. However, Shamsuzzaman and colleagues showed that VDR deficiency in an ApoE^−/−^ background combined with high fat diet (HFD) feeding protected mice against hypercholesterolemia-induced VC, even though they did develop atherosclerotic lesions [47]. A third study by Han et al. treated VDR^−/−^ mice with a high dose of vitamin D and demonstrated that they were protected against vitamin D-induced VC [48]. The fact that under conditions that normally lead to VC (i.e., hypercholesterolemia or supraphysiological vitamin D levels) VDR absence prevents VC development has shed some doubts on the results from the study of Schmidt and co-workers. It has been mentioned that the calcification was only observed on the valve leaflets where pigmentation may be mistakenly identified as calcification by von Kossa histochemical stain [49]. However, the observed increase in aortic expression of osteogenic genes still would be in line with increased calcification. In this context, it should be pointed out that humans with vitamin-D-dependent rickets type 2A, that have loss-of-function mutations in VDR, are not reported to suffer from VC, providing another argument to conclude that VDR absence protects against VC [50]. It would be interesting to use conditional VDR knockout models to decipher the role of VDR in the individual cell types that are implicated in VC. In this respect, it is important to note that one study transplanted aortas from VDR^−/−^ mice into wild-type mice, before induction of uremia and treatment with vitamin D, and no differences in aortic calcification was observed between VDR^−/−^ aortic allografts and VDR^+/+^ recipient aortas, suggesting that VDR activation promoted VC through a systemic action rather than through a direct vascular action [51]. Similarly, it would be interesting to study the effects of (conditional) overexpression of VDR on VC. Taken together, mouse and human data seem to show that VDR absence is protective of VC but the specific role of VDR actions in particular cell types and/or tissues in development of VC remains to be established.

### 2.2. Human Steroid and Xenobiotic Receptor (SXR) and Its Rodent Homolog Pregnane X Receptor (PXR)

Similar to vitamin D, vitamin K deficiency has been recognized as another important risk factor for the development of VC [52]. Vitamin K exists in two natural forms: K1 or phylloquinone (PK) found in vegetables, and K2 or menaquinones (MKs) derived from bacteria and fermented food. MK4 and MK7 are the most extensively studied vitamers of the vitamin K2 family and MK4 can be metabolized from PK. Vitamin K is best known for its role as the co-enzyme for the vitamin-K-dependent carboxylase that converts specific glutamic acid (Glu) residues on so-called vitamin-K-dependent proteins (VKDPs) into calcium-binding carboxyglutamic acid (Gla) residues. Several of these VKDPs are involved in blood coagulation but others are known to improve bone and vascular health, including bone Gla protein (BGP or osteocalcin) and matrix Gla protein (MGP). The latter is secreted by VSMCs and inhibits VC through poorly studied mechanisms [53]. These findings support a protective role for vitamin K in VC and this has been confirmed by numerous other studies [54,55,56,57,58,59,60,61,62].

In line with such a protective role of vitamin K in VC, its antagonist warfarin, a staple in anticoagulant therapy, has been shown to promote VC by several mechanisms, including inhibition of vitamin-K-dependent carboxylation of MGP [63]. Another mechanism of warfarin-induced VC could involve the steroid and xenobiotic receptor (SXR, rodent homolog is pregnane X receptor (PXR)). Warfarin was previously shown to be able to interact with SXR/PXR [64] and warfarin-induced calcification of human aortic valve interstitial cells (HAVICs) could be inhibited by (non-specific) SXR inhibitors ketoconazole and coumestrol [65]. This mechanism could involve warfarin-induced increase in expression of transglutaminase 2 (TG2) through SXR/PXR and involves β-catenin signaling [66]. Interestingly, the selective PXR agonist SR-12813 also induced HAVIC calcification [65]. This suggests that other ligands of SXR/PXR could also induce HAVIC calcification. In line with this, MK4, which has also been shown to be a ligand of SXR/PXR [67,68], was shown to dose-dependently accelerate HAVIC calcification [69]. MK4 and warfarin exhibit structural similarity and were therefore thought to compete for the same binding site on SXR/PXR. However, surprisingly, MK4 seems to accelerate warfarin-induced HAVIC calcification [69]. Warfarin, SR-12813, and MK4 were all three shown to induce expression of BMP-2 (a calcification inducer of the bone morphogenetic proteins, members of the transforming growth factor β family) leading to enhanced TNAP/ALP activity [65,69]. This pro-VC role of MK4 does not match with the abovementioned protective role of vitamin K in VC. However, genes induced by vitamin K in an SXR/PXR-dependent manner included osteogenic markers (e.g., TNAP/ALP, osteoprotegerin (OPG), osteopontin (OPN), and MGP) and ECM proteins involved in collagen accumulation [67,70]. In line with this, PXR knockout mice display osteopenia with reduced bone formation and enhanced bone resorption [71]. Considering the inverse relationship between bone resorption and VC, it would be interesting to know whether PXR knockout mice suffer from increased VC, but this has not been reported or studied to our knowledge. Interestingly, SXR/PXR can also interact with vitamin D metabolism. SXR/PXR activation can lead to increased CYP3A4 expression and this enzyme can metabolize vitamin D through its 24-hydroxylase activity, leading to vitamin D deficiency. SXR activation can also lead to inhibition of 24-hydroxylase activity in the kidney and thereby increase vitamin D levels [72]. Taken together, SXR/PXR can play a role in VC as a receptor for warfarin and/or vitamin K and/or through crosstalk with vitamin D metabolism. For now, the limited data available does not allow for a clear-cut conclusion as to whether SXR/PXR activation is protective in the context of VC or not. Additional in vitro and in vivo studies using specific SXR/PXR (ant)agonists, in combination with (conditional) knockout/knockdown studies, are required to delineate the precise role of SXR/PXR in VC.

### 2.3. Sex Hormones and Receptors: Estrogen Receptors (ER), Androgen Receptor (AR) and Progesterone Receptor (PrR)

#### 2.3.1. Estrogen Receptors

The estrogen receptors (ERα and ERβ) signal through similar cell pathways but differ in both transcriptional activity and regulation [73]. Hence, tissue-specific ER expression and localization may affect the vascular response.

Estrogen regulates bone metabolism by inducing OTC apoptosis [74] and OTB expression of OPG [75]. In the controlled Women’s Health Initiative Study, postmenopausal women who had undergone hysterectomy, treated with long-term estrogen therapy had lower coronary artery calcium score than those receiving placebo [76]. Similar results were obtained in another clinical study including postmenopausal women showing that estrogen replacement significantly lowered calcium score [77]. Analysis of coronary arteries from postmenopausal women treated with estrogen revealed that calcium and plaque area were strongly correlated with estrogen status after correction for coronary heart disease, thus suggesting an anti-atherosclerotic effect of estrogen use [78]. Additionally, postmenopausal women with higher serum 17β-estradiol (E2) levels had a reduced coronary artery calcification independent of age and other coronary risk factors, thus suggesting that higher level of E2 can possibly reduce VC [79]. In a multicenter randomized placebo-controlled trial in early menopausal women, the effects of oral conjugated equine estrogens and transdermal E2 administration were tested compared to placebo [80]. Transdermal E2 administration increased coronary artery calcification, associated with accumulation of paracardial adipose tissue, which is located anterior to the epicardial adipose tissue [80]. These results underscored the different contributions of estrogen type and route of administration in assessing their effects. Globally, these findings are supported by data from animal studies showing that E2 protects against atherosclerotic plaque calcification when associated with beneficial effects on plaque progression [81]. Treatment of Western diet-fed ovariectomized ApoE^−/−^ mice (presenting both advanced atherosclerotic calcification and osteoporosis) with E2 inhibited osteoporosis and the BMP osteogenic pathways in aortas by decreasing SMAD1/5/8 phosphorylation, thus leading to reduction in calcium accumulation [82]. Estrogen deficiency in female castrated rats enhanced vascular calcification, whereas E2 administration reduced the hypoxia-induced factor 1 alpha (HIF-1α) and vascular calcification in rats [83]. Administration of raloxifene, a selective ER modulator, to cholesterol-fed ovariectomized rabbits did not modify the total vascular calcification, compared to untreated rabbits, but calcifications were characterized by less nodular and better radial organization, as predicted by an increased expression of BMP-2, thus enhancing mechanical stability of calcifications [84]. However, the mechanistic similarities to bone formation suggest that E2 could promote VC in certain circumstances. Treatment of both male and female ApoE^−/−^ mice with silastic E2 capsule implantation increased calcified area in the aortic sinus, but not in brachiocephalic arteries, independently of the effects on plaque growth or lipid levels and occurred with a reduction in the proportion of ERβ-positive intimal cells, without affecting the proportion of ERα-positive cells [85]. This was accompanied by reduced expression of the mineralization inhibitor, MGP, but increased expression of the osteogenic markers, bone sialoproteins (BSP) and collagen II [85]. These results continue to raise the question of how estrogens impact on VC and plaque progression in the longer term, particularly in the aging vasculature [86], where established, more advanced lesions show a reduced anti-atherosclerotic response to exogenous estrogen treatment in females [87]. Indeed, the direct role of ERs in the effects observed on VC upon E2 treatment are far from being completely elucidated. Both ERα and ERβ were expressed in arterial VSMC, but ERβ was the predominant ER form in the intima of coronary artery in women and correlated with calcium content and with the calcium/plaque area ratio [88]. Increased intimal ERβ expression was linked to advanced atherosclerosis and calcification, independently of age or hormone status [88]. However, expression of ERα did not correlate with calcium content nor with the calcium/plaque area ratio [88]. In vitro experiments investigating the role of estrogens and ER in cell mineralization led to contradictory results. The first experimental evidence of a potential role of estrogens in VC came from the observation that treatment of bovine aortic medial cells with E2 significantly increased cell calcium content, formation of calcified nodules and induced the activity of TNAP/ALP and secretion of osteocalcin [89]. In vitro, treatment of bovine coronary artery VSMC with E2 suppressed ERβ expression and increased cell mineralization, as demonstrated by increasing expression of collagen I and II, osteocalcin and BSP, and by reducing MGP and OPN. Antagonism or silencing of ERα, ERβ or both further increased VSMC mineralization [85]. However, other studies have demonstrated an inhibitory role for E2 and ERs in VSMC calcification. Indeed, E2, as well as raloxifene, reduced inorganic phosphate (Pi)-induced calcification and preserved the contractile phenotype of VSMC isolated from gonadally intact or ovariectomized female pigs. E2 and raloxifene decreased expression of OPG, an effect that was significantly greater in ovariectomized compared to gonadally intact pigs, while expression of BSP was inhibited in cells from both groups [90]. The effects of E2 have also been evaluated in human aortic endothelial cells (HAECs) and human aortic SMCs (HASMCs). The ERα mRNA level was higher than ERβ in HASMCs, and they were expressed at the same level in HAECs and OTBs [82]. E2 inhibited the RANKL-induced BMP-2 as well as the RANKL-decreased expression of MGP (calcification inhibitor) in HAECs and in HASMCs, respectively [82]. The effects of E2 were mainly mediated by ERα since they were lost in the presence of the ERα inhibitor ICI-182,780. Moreover, treatment of human HUVEC cells with both E2 or raloxifene significantly lowered OPG concentration both in basal as well as in calcifying medium, without affecting RANKL expression [91]. E2 treatment of rat VSMC [83] decreased calcium concentration as well as calcium nodule formation by affecting the protein levels of BMP2-pSMAD1/5/8 via reducing the expression of HIF-1α, the latter playing an important role in VC [92]. Another potential protective effect of estrogens against VC has been identified. Indeed, E2 treatment of HASMC at physiological concentrations inhibited the Pi-induced calcification in a specific ERα-dependent manner [93]. Apoptosis, which is an essential process for VSMC calcification, was inhibited by E2 and addition of MPP (an ERα selective antagonist) abolished this inhibition. From a mechanistic point of view, E2 transactivated, through ERα, the promoter of Gas6 (growth arrest-specific gene 6), a key molecule regulating VSMC calcification through apoptosis [94]. E2 increased Pi-downregulated Gas6 and phospho AKT expression, thus suggesting that E2 restored the Gas6-mediated survival pathway.

Taken together, these data underline a complex role for estrogens in the control of VC which is highly dependent on the cell model used for mechanistical in vitro studies as well as on the post-menopausal status of treated patients and animal models for in vivo studies.

#### 2.3.2. Androgen Receptor

Androgens have significant effects on bone formation via regulation of both osteoblast and osteoclast cell types [95]. Moreover, since VC is more prevalent in men, this suggests a potential influence of male hormones [96]. Immunohistochemical analyses revealed the presence of androgen receptor (AR) in the calcified media of human femoral artery and calcified human valves [97]. However, in a clinical analysis, no association between coronary artery calcification in men and AR expression was observed [98]. Potentially, the exclusion of patients with pre-existing coronary artery disease may have underestimated any association between AR expression and calcification in that study. Indeed, administration of testosterone and dihydrotestosterone (DHT, a non aromatizable androgen) in gonadally intact male and female ApoE^−/−^ mice led to an increased calcification in the brachiocephalic artery, while only testosterone had an effect on aortic sinus [99]. These effects were independent of mouse sex and occurred despite corresponding reduction of plaque area, the latter correlating with an increase in high density lipoprotein (HDL) concentrations. Androgen-induced calcification of the brachiocephalic artery was paralleled by an increased expression of AR upon administration of testosterone and DHT both in male and female mice, without affecting the expression of ERα nor ERβ in either sex. Conversely, in aortic sinus, induction of calcification by testosterone was accompanied by a downregulation of ERα but not ERβ in both sexes, while expression of AR was increased in female but not in male mice. This indicates that calcification is a process sensitive to androgens with effects that are independent from those observed on plaque reduction and cholesterol levels. However, the effects of testosterone on vascular calcification are quite controversial, possibly due to the complex in vivo effect of this hormone. Indeed, low testosterone levels were associated with an increase of aortic calcification in elderly men [100].

In vitro, testosterone and DHT treatment increased Pi-induced mouse VSMC calcification by increasing TNAP/ALP mRNA expression, an effect blunted in AR-deficient VSMC [97]. However, protective potential mechanisms of androgens have been identified. Indeed, testosterone and DHT inhibited the Pi-induced calcification of human aortic VSMC by preventing the cell apoptosis, an essential process for calcification, by restoring the Pi-downregulated expression of Gas6. These effects of androgens were blocked by an AR inhibitor, flutamide, but not by ICI 182.780, an antagonist of ER. AR transactivated Gas6 promoter activity through two AREs (androgen response element) consensus sites located in the Gas6 proximal promoter [101]. AR-mediated Gas6 transactivation was also observed in the inhibitory effects on VSMC calcification by Ginsenoside Rb1, a selective AR modulator [102]. The discrepancies between these different published studies may reflect key differences in the species, passage status and culture conditions of the VSMC model used (mouse vs human). Moreover, the role of macrophage AR on Pi-induced VSMC calcification has also been investigated [103]. Conditioned medium from AR silenced THP-1 cells inhibited the Pi-induced VSMC calcification by reducing the protein expression of Runx-2, an OTB marker, and by increasing the expression of SM22a, a SMC marker. The role of macrophage AR on SMC calcification was related to a reduced production of IL-6 in the silenced cells.

#### 2.3.3. Progesterone Receptor (PgR)

VSMCs were shown to express PgR [104] and both progesterone (Pg) and the synthetic progestin medroxyprogesterone (MPA) reduced VSMC osteogenic-like transdifferentiation as demonstrated by a significant reduction of TNAP/ALP activity and ECM mineralization [105]. Besides this anti-osteogenic action on bone cells, the progestogens induced OTB maturation and mineralization. Both these Pg actions could be inhibited by the PgR antagonist RU486, indicating the involvement of PgR in these Pg-regulated processes. Together, these results show that PgR activation could have beneficial effects both in the context of VC and osteoporosis.

### 2.4. Peroxisome Proliferator-Activated Receptors (PPAR)

PPARs are lipid-activated NRs regulating lipid and glucose metabolism in metabolic tissues, such as the adipose tissue and the liver [106]. They are also expressed in cells of the vasculature (endothelial cells, SMC, monocytes/macrophages and lymphocytes), where they mainly control the inflammatory response [107]. Among the three members of the PPAR family, PPARα, PPARβ/δ and PPARγ, only the latter has been largely studied in the context of VC. Accelerated VC was observed in mice lacking PPARγ selectively in the VSMC (SM22Cre+/PPARγ^flox/flox^ mice in a LDL-receptor-deficient (LDLR^−/−^) background, smPPARγ− mice) [108]. mRNA levels of chondro-osteogenic factors promoting mineralization, such as the Fos-related AP1 transcription factor (Fra2), collagens 10 A1 and 2A (Col10a1, Col2a), osteocalcin, TNAP/ALP and Runx-2, were significantly increased in aortas of smPPARγ− mice. Moreover, gene expression of Wnt5a, a promoter of chondrogenic differentiation, was also increased. Interestingly, a positive Wnt5a immunoreactivity was observed in human carotid atherosclerotic lesions where Wnt5a mRNA increased in calcified regions [108]. PPARγ protected against calcification by inducing the expression of secreted frizzled-related protein 2 (sFRP2), a Wnt5a antagonist [109]. The VC in the absence of PPARγ depended on the expression of the trans-membrane LDLR-related protein 1 (LRP-1), shown to be required for a Wnt5a-dependent pro-chondrogenic pathway. Indeed, LDLR^−/−^ mice specifically deficient for both PPARγ and LRP1 in VSMCs had no accumulation of calcium depots in their atherosclerotic lesions. When treated with a chondrogenic cocktail, VSMCs isolated from aortas of these mice were resistant to differentiation [108]. The involvement of Wnt/β-catenin pathway in the effects of PPARγ activation in calcification has been also proven [110]. Treatment of rat calcified VSMC with pioglitazone, a synthetic PPARγ ligand, reduced the extracellular calcium accumulation as well as the calcification-induced protein expression of β-catenin, p-GSK-3β and cyclin D1, by a PPARγ-dependent mechanism. Another possible mechanism by which PPARγ controls VC has been identified [111]. Deficiency of Klotho, encoding a single span transmembrane protein, primarily expressed in renal tubular epithelial cells [112], promoted calcification and osteoblastic differentiation of VSMCs [113]. Treatment of bovine aorta VSMCs with the PPARγ agonist rosiglitazone inhibited the Pi-induced calcification by enhancing the expression of Klotho in a PPARγ-dependent manner. Interestingly, reduction of Klotho expression by RNA interference abolished the ability of PPARγ activation to inhibit VSMC calcification, indicating that PPARγ regulated calcification by a Klotho-dependent mechanism [111]. The relationship between PPARγ and Klotho in calcification has been further confirmed [114]. Indeed, rosiglitazone failed to reduce Pi-induced calcification in VSMC with siRNA-silenced expression of Klotho as well as in the aortic rings from Klotho-deficient mice [114]. Interestingly, Pi-induced calcification led to a reduced expression of PPARγ protein and mRNA [111,114] by an epigenetic mechanism involving the methyl-Cpg binding protein 2 (Mecp2) [114], as confirmed by the fact that Mecp2 siRNA reversed the decreased expression of PPARγ after Pi-induced calcification. This agrees with the observation that PPARγ protein expression decreased in the calcified radial arteries of CKD patients, accompanied by an increased expression of osteogenic factors (Runx-2, BMP-2), while the expression of Klotho increased [114]. Since VC is a common feature in patients with diabetes mellitus, and the elevated glucose observed in such patients may directly affect the calcification process by modifying VSMC phenotype [115], the role of PPARγ has been studied in vitro under hyperglycemic conditions [116]. VSMC isolated from rat aorta cultured under high glucose concentrations (HG, 25 mM) showed a decreased expression of PPARγ protein and mRNA, which was restored by the addition of the PPARγ ligand rosiglitazone. HG also induced calcium deposition and increased TNAP/ALP activity and OTB-like phenotype in calcified VSMCs. Addition of troglitazone and rosiglitazone attenuated VSMCs calcification under HG conditions by downregulating Runx-2, osteocalcin, and BMP-2 expression and by upregulating the mineralization inhibitor MGP. The presence of the PPARγ antagonist (GW9662) blocked the effects of rosiglitazone, thus underlying the direct PPARγ protective effects against calcification. Unsaturated N-3 fatty acids, cis–5,8,11,14,17-eicosapentaenoic acid (EPA) and cis–4,7,10,13,16,19-docosahexaenoic acid (DHA), potent natural anti-inflammatory PPAR-γ agonists [117], inhibited both spontaneous and IL-6-induced OTB differentiation in calcifying vascular cells by a mechanism dependent on the p38-mitogen-activated protein kinase (MAPK) [118]. Moreover, known PPARγ agonists (troglitazone and ciglitazone) inhibited cell mineralization to an extent comparable to those obtained by DHA, suggesting that activation of PPARγ mediates DHA inhibitory effect, even though direct evidence was not provided.

### 2.5. Liver X Receptors (LXR)

LXRα and LXRβ are NRs mainly activated by oxysterols [119]. LXRα is significantly expressed in metabolically active tissues, such as the liver, whereas LXRβ is ubiquitously present. The effects of LXR on VC have been studied both in vitro and in vivo. In vitro, LXR activation by T0901317, a specific ligand, increased protein kinase A (PKA)-induced mineralization in aortic VSMC isolated from control mice, but not from LXRβ-deficient mice, thus indicating the specific involvement of LXRβ in this process [120]. LXR activation enhanced PKA-stimulated mineralization by positively regulating the activity and expression of mineralization inducers, such as TNAP/ALP and phosphate transporter Pit-1, respectively, as well as by attenuating the expression of mineralization inhibitors (OPN and ectonucleotide pyrophosphate/phosphodiesterase-1 (Enpp1)) [120]. These results have been further confirmed and extended. Indeed, activation of LXR by synthetic ligands (T0901317 and GW3965), or by adenovirus overexpression of constitutively activated forms of both LXRα and LXRβ, accelerated mineralization of bovine calcifying vascular cells [121]. Inhibition of LXR activity by dominant negative forms of LXRα and LXRβ reduced calcium content in these cells. The effects of LXR agonists on calcification have been correlated with lipid accumulation, fatty acid synthesis and expression of sterol regulatory element binding protein 1 (SREBP-1c). Cellular SREBP-1c dependent lipogenesis was increased by LXR activation, leading to a cellular accumulation of stearate, shown to markedly promote cell calcification compared to other fatty acids. SREBP-1c overexpression increased cell mineralization, whereas SERBP-1c inhibition blocked TNAP/ALP activity and mineralization induced by LXR activation [121]. More recently, pharmacological approaches have been developed to specifically target macrophage LXR but not hepatocyte LXR, thus avoiding induction of hepatic lipogenesis [122]. Subcutaneous injection of nanofiber hydrogel containing T0901317 in ApoE^−/−^ mice submitted to high fat diet (HFD) resulted in a significant reduction of calcium depots (microcalcifications) in the established atherosclerotic lesions, to an extent comparable to those obtained by oral administration of T0901317 [122]. However, no mechanistic data were provided to explain the effect of the nanofiber containing T0901317 on VC, these in vivo data appear contradictory to the previously published results based on in vitro experiments, suggesting a pro-calcifying role for LXR. Altogether, these results indicate that LXR activation controls VC probably by multiple mechanisms going from the regulation of the expression of osteogenic genes to the generation of fatty acid metabolites involved in the mineralization process. An involvement of tissue specific LXR action and/or specific effects of LXRα or LXRβ activation can also be hypothesized.

### 2.6. Farnesoid X Receptor (FXR)

FXR is an NR activated by bile acids (BAs), the most potent endogenous ligand being chenodeoxycholic acid; it regulates BA, lipid (cholesterol) and glucose homeostasis, and also has a regulatory role in inflammation and mitochondrial function [123]. FXR is not only highly expressed in the liver, kidney and intestine but is also present in cell types of the vascular wall, including VSMC and endothelial cells [124,125]. FXR activation by the synthetic FXR ligand 6α-ethyl chenodeoxycholic acid (INT-747) and by adenovirus-mediated overexpression of a constitutively active form of FXR blocked mineralization and lipid accumulation of bovine calcifying vascular cells (CVCs) in response to Pi, while a dominant negative form of FXR augmented mineralization of CVC and blocked the anti-calcific effect of INT-747. In addition, treatment with INT-747 inhibited VC in ApoE^−/−^ mice with CKD without impacting atherosclerosis development [126]. Several mechanisms have been proposed for this protective effect of FXR activation in VC. Since INT-747 treatment increased phosphorylated c-Jun N-terminal kinase (JNK) and treatment with SP600125 (specific JNK inhibitor) significantly induced mineralization of CVC and TNAP/ALP expression, it was suggested that the anti-calcific effect of INT-747 was due to JNK activation [126]. A more recent study showed that FXR activation increased miR-135a-5p expression, which inhibited the activation of the transforming growth factor-β receptor 1 (TGFBR1)/TGF-β-activated kinase 1 (TAK1) pathway, ultimately resulting in the attenuation of vascular inflammation and calcification in CKD rats [127]. Activation of the TGFBR1/TAK1 pathway in HASMCs by an osteogenic medium was inhibited by obeticholic acid (OCA, a FXR agonist) and this was accompanied by a reduction in NF-κB and TNFα expression and attenuated calcification. OCA also inhibited the expression levels of Runx-2 and TNAP/ALP. In vivo, OCA-mediated FXR activation retarded novel formation of VC but did not reverse already established VC. These OCA effects could be partially abolished by a miR-135a-5p inhibitor. Together, the data suggested that TGFBR1 is a direct target of miR-135a-5p and that OCA-mediated FXR activation upregulated miR-135a-5p expression, thereby inhibiting the TGFBR1/TAK1 pathway. Lastly, another recent study showed that treatment of cultured VSMCs with deoxycholic acid (DCA, but no other BAs) induced osteogenic differentiation and calcification through endoplasmic reticulum (ER) stress-mediated ATF4 activation. Treatment of mice with FXR-specific agonists selectively reduced levels of circulating cholic acid (CA)-derived BAs such as DCA, protecting from CKD-dependent medial calcification and atherosclerotic calcification. Reciprocal FXR deficiency and DCA treatment induced VC by increasing levels of circulating DCA and activating the ER stress response [128]. Taken together, these results suggest that FXR activation could serve as a therapeutic strategy for retarding VC in CKD patients. Interestingly, in bone marrow stromal cells, FXR activation increased calcification [129], suggesting that in the context of the inverse relationship between osteoporosis and VC, FXR activation would lead to a win-win situation.

### 2.7. Mineralocorticoid Receptor (MR) and Glucocorticoid Receptor (GR)

#### 2.7.1. Mineralocorticoid Receptor (MR)

MR has been shown to be expressed in endothelial cells and VSMCs in the vessel wall [130]. Different ligands for MR have been identified. Cortisol and aldosterone bind to human MR with similar affinities [131]. However, the enzyme 11β-hydroxysteroid dehydrogenase type 2 (11βHSD2) converts cortisol to cortisone and the latter has low affinity for MR [132]. Co-expression of MR and 11βHSD2 in vascular expression might therefore exclude the activation of MR by cortisol in the vasculature and this mechanism would confer MR selectivity to aldosterone [133]. This remains however a matter of debate since the enzyme 11β-hydroxysteroid dyhydrogenase type 1 (11βHSD1) can regenerate cortisol from cortisone and murine VSMCs do not appear to express 11βHSD2 [132,134]. Taken together, it remains to be elucidated whether cortisol or aldosterone might be the more prominent ligand of MR on VSMCs depending on local 11βHSD2-11βHSD1 activity and/or local aldosterone/cortisone synthesis and context of disease [135]. Furthermore, angiotensin II (Ang II) has also been shown to be able to activate MR through the Ang II type 1 receptor [136].

MR activation by aldosterone has been shown to promote the expression of genes involved in VC in VSMCs, such as TNAP/ALP and BMP-2 and promoted cell mineralization [137]. This could be inhibited by the MR antagonist spironolactone (SPL). The same MR antagonist has been shown to prevent VC in CKD rats and in Klotho hypomorphic mice [138,139]. This SPL-induced attenuation of VC involved the downregulation of the type III sodium-dependent phosphate co-transporter 1 (Pit-1), required for Pi-induced calcification of VSMCs [140]. A recent study suggested that aldosterone facilitated high Pi-induced VSMC calcification through an MR-involved AMPK-dependent autophagy [141]. Moreover, another study showed that miR-34b/c participates in aldosterone-induced VSMC calcification [142]. Regarding the involvement of MR in corticosterone-induced VC, one study showed that while a glucocorticoid receptor (GR) antagonist (mifepristone) had no effect on mouse VSMC calcification, the MR antagonist eplerenone decreased corticosterone-induced VSMC calcification [134]. However, the latter study did not observe the increase in osteogenic genes observed with aldesterone-induced VSMC calcification but instead it suggested VSMC apoptosis as an alternative mechanism contributing to calcification. Apoptosis can promote calcification by effects that include release of apoptotic bodies with precipitation of hydroxyapatite [143]. While the above-described in vitro studies demonstrate a role for VSMC MR expression in VC, an in vivo study using conditional SMC-MR knockout mice in an ApoE^−/−^ background claimed that there is no significant role for SMC MR in VC [144]. However, there was already barely any VC observed in the control ApoE^−/−^ littermates which might explain the lack of an observed effect of conditional SMC MR deficiency. It would be more interesting to study the effect of SMC MR deficiency in a model that is more prone to VC (e.g., aldosterone administration). Overall, the available data attribute a positive role for MR in VC suggesting that MR inhibitors are interesting pharmaceutical candidates in the prevention/treatment of VC.

#### 2.7.2. Glucocorticoid Receptor (GR)

Dexamethasone, a ligand of GR, has been shown to enhance osteogenic differentiation in bovine VSMCs and vascular pericytes [145,146]. In the latter cells, this could be blocked by the GR agonist Org 34116, proving the implication of GR, while in the VSMCs the involvement of GR was not studied. Moreover, GR deficiency in macrophages led to reduction in VC in LDLR^−/−^ mice while not changing atherosclerotic lesion size [147]. This study also showed that conditioned media from dexamethasone-treated macrophages can induce calcification in VSMCs, suggesting that macrophage GR activation can indirectly stimulate calcification in vascular cells. It was speculated that this paracrine effect may be partially mediated by GR signaling-induced apoptosis in macrophages [148]. In contrast, U-74389G, a non-glucocorticoid steroid thought to be devoid of GR activity, was shown to attenuate IL-1β-induced calcification of HAVICs [149]. This anti-osteogenic activity could be blocked by the GR antagonist mifepristone, suggesting that these actions were mediated by GR. Overall, the specific role of GR in VC still remains to be elucidated since most data come from in vitro studies and, as discussed above in the part concerning the MR, the specific role of GR in glucorticoid regulation of VC has been questioned [134].

### 2.8. Retinoic Acid Receptor (RAR)

There have been contradictory reports on the effect of the active vitamin A metabolite all-trans retinoid acid (ATRA), a ligand of RAR, on ectopic calcification. Treatment of mice heart valve leaflets with ATRA in vitro induced calcification [150] and increased dietary intake of vitamin A promoted aortic valve calcification in vivo in mice [151]. However, other studies found that the synthetic selective RARγ agonist NRX204647 could inhibit vascular calcification [152] and ATRA decreased vitamin D-induced renal calcification in mice [153]. More recently, it was demonstrated that treatment with ATRA, as well as an acyclic synthetic retinoid, peretinoin, reduced calcification and osteogenic differentiation of primary human coronary SMCs and valve interstitial cells [154]. This involved a reduced expression of both TNAP/ALP and Runx-2 and an increase in MGP. Furthermore, this inhibitory effect could be neutralized by siRNA-mediated knockdown or inhibition of RAR (with antagonist AGN 193109), with both approaches increasing calcification. Interestingly, ATRA was shown to also reduce calcification in human osteoblasts while the acyclic retinoid peretinoin did not, suggesting that acyclic retinoids may allow for treating VC without adverse effects on bone homeostasis. Nonetheless, an overall conclusion on the role of RAR in VC requires more studies, with a specific focus on potential species differences.

### 2.9. Orphan Nuclear Receptors

Very few data are available concerning the control of VC by the family of orphan nuclear receptors. Among them, estrogen-related receptor (ERR)γ has been studied. ERRγ, whose expression was induced in calcified VSMC, induced the expression of osteogenic genes Runx2, OPN, Msx2 and BMP-2, leading to increased phosphorylation of the BMP-2 effector proteins, SMAD1/5/8 [155]. ERRγ inhibition by specific siRNA or inverse agonist GSK5182 attenuated VC and inhibited the expression of osteogenic markers both in vitro and in vivo in mice injected subcutaneously with cholecalciferol (vitamin D_3_), by reducing the proportion of BMP-2 positive areas [155].

The other orphan nuclear receptor investigated in the context of VC is NR4A1, also named Nur77 [156]. Protein expression of NR4A1 was higher in rat calcified aorta compared to non-calcified tissues, in a VC model obtained by intravenous injection of vitamin D_3_ and nicotinamide, an effect amplified by lactate administration [157]. In vitro studies provided evidence that NR4A1 was involved in the lactate-induced calcification of rat VSMC [157]. Indeed, using NR4A silencing or induction by cytosporone B (CsnB), it was reported that lactate enhanced mitochondrial fission but suppressed mitophagy, two processes involved in VC, via activation of the NR4A1/DNA-PKcs/p53 pathway, leading to apoptosis and accelerating the OTB phenotype transition of VSMC and calcium deposition.

The potential involvement of other orphan nuclear receptors (e.g., NOR-1, Nurr1, RORs) in the process of VC is unknown.

## 3. Conclusions and Perspectives

From the 13 NRs for which we discuss published findings on their role in VC (summarized in Table 1) we could not draw firm conclusions due to contradictory reports on 5 of them (i.e., RAR, SXR/PXR ER, AR, and LXR). In the case of RAR this could be due to species differences and for LXR in vivo studies seems to contradict in vitro data. For 5 out of the 13 NRs we concluded that their activation would induce VC (i.e., VDR, ERRγ, NR4A1, MR and GR), although for GR the physiological relevance is unclear. Furthermore, for ERRγ and NR4A1 we found only one study each, and thus this needs to be confirmed. Three out of the thirteen NRs seem to have a protective role in VC (i.e., PPARγ, FXR, and PgR). While several studies have solidified this conclusion for PPARγ and FXR, the PgR data were from a single study and also need confirmation. Table 2 shows how different NRs regulate genes/proteins involved in VC. The conflicting reports of the role of ER in VC is apparent in this table with contradictory effects described for ER on BMP2, MGP and OPG. The precise role of many of these NRs in VC remains to be elucidated. Most data come from in vitro studies and (conditional) and/or cell-type- or tissue-specific knockout or overexpression animal models of these NRs would be of great value to define their precise role in VC. Specifically, the role of NR expression in inflammatory cells such as macrophages would be worth evaluating. Furthermore, clinical data are even rarer and are highly needed. Overall, this leads us to conclude that for now, based on our current knowledge, the most attractive therapeutic NR targets to prevent or treat VC will be by activating PPARγ or FXR, or by inhibiting MR. In the ideal case, such treatments should inhibit both VC and osteoporosis.

## Figures and Tables

**Table 1:** List of NRs and their roles in VC.

NR	Short Description of Findings	+ or − Role *	Refs
VDR	U-shaped dualistic role for vitamin D; both low and high levels linked to VC. Contradictory VDR^−/−^ mouse studies but mostly supporting inducing role. Human data: VDR mutations do not lead to VC.	+	[43,44,45,46,47,48,50]
SXR/PXR	Several studies support a protective role for vitamin K in VC. However, MK4 can induce calcification through SXR/PXR. PXR^−/−^ mice have osteopenia but VC was not studied. Crosstalk SXR/PXR and vitamin D metabolism. No clear-cut conclusion regarding role SXP/PXR in VC.	?	[54,55,56,57,58,59,60,61,62,67,69,70,71,72]
ER	Rodent and human studies demonstrate protective role for estradiol in VC. However, other rodent studies show opposite. Contradictory in vitro results.	?	[76,77,78,79,81,82,83,84,85,89,90,91,93]
AR	Androgen treatment was shown to induce VC but low androgen has been associated with increased VC. Contradictory in vitro effects.	?	[97,99,100,101,102]
PgR	In vitro studies show that PgR activation has beneficial effects for both VC and osteoporosis.	−	[105]
PPARγ	SMC knockout leads to increase in VC. Involves LRP1-Wnt pathway and other mechanisms through Klotho.	−	[108,109,110,111,113,114]
LXR	Activation induces mineralization in vitro. However, in vivo studies show the opposite.	?	[120,121,122]
FXR	In vitro and in vivo data show that FXR activation leads to a decrease in VC. Multiple mechanisms proposed.	−	[126,127,128]
MR	Not sure whether aldosterone or cortisol activates MR in vasculature. MR activation by aldosterone leads to increase in VC. MR inhibitors can potentially prevent/treat VC.	+	[132,133,134,135,137,138,139,141,142]
GR	GR activation leads to increased VC. Opposite results with non-glucocorticoid steroid that binds to GR. Still, natural ligand leads to increased VC but in vivo implication not sure.	+?	[134,145,146,149]
RAR	Contradictory in vitro and in vivo results. In mice it induces VC while in humans it inhibits.	?	[150,151,152,153,154]
Orphan	ERRγ: Activation increases VC in vitro and in vivo. NR4A1: Activation increases VC.	+	[155] [157]

* + indicates that activation of this NR leads to induction of VC. − indicates that activation of this NR leads to inhibition of VC. ? indicates that role is unclear.

**Table 2:** List of genes/proteins involved in vascular calcification and how they are regulated by NRs.

Gene/Protein Involved in Vascular Calcification	NRs with Inducing Effect	NRs with Reducing Effect
TNAP/ALP	SXR/PXR [65,69,70]	PgR [105] PPARγ [108]
	ER [89] AR [95,97] LXR [120]	FXR [126]
	MR [137]	RAR [154]
BMP2	SXR/PXR [65,69] ER [84]	ER [82]
	MR [137] ERRγ [155]	PPARγ [114]
Runx-2	ERRγ [155]	PPARγ [108,114]
		FXR [127] RAR [150]
Osteocalcin	ER [89]	PPARγ [108]
MGP	SXR/PXR [67,70] ER [82]	ER [85]
	PPARγ [116] RAR [154]	
OPN	SXR/PXR [67,70]	ER [85]
	ERRγ [155]	LXR [120]
OPG	SXR/PXR [67,70]	ER [90,91]
	ER [75]	
